# Optical Multi-Parameter Measuring System for Fluid and Air Bubble Recognition

**DOI:** 10.3390/s23156684

**Published:** 2023-07-26

**Authors:** Valentina Bello, Elisabetta Bodo, Sabina Merlo

**Affiliations:** Department of Electrical, Computer and Biomedical Engineering, University of Pavia, 27100 Pavia, Italy; valentina.bello01@universitadipavia.it (V.B.); elisabetta.bodo01@universitadipavia.it (E.B.)

**Keywords:** air bubbles, laser beam, optical sensor, position sensitive detector

## Abstract

Detection of air bubbles in fluidic channels plays a fundamental role in all that medical equipment where liquids flow inside patients’ blood vessels or bodies. In this work, we propose a multi-parameter sensing system for simultaneous recognition of the fluid, on the basis of its refractive index and of the air bubble transit. The selected optofluidic platform has been designed and studied to be integrated into automatic pumps for the administration of commercial liquid. The sensor includes a laser beam that crosses twice a plastic cuvette, provided with a back mirror, and a position-sensitive detector. The identification of fluids is carried out by measuring the displacement of the output beam on the detector active surface and the detection of single air bubbles can be performed with the same instrumental scheme, exploiting a specific signal analysis. When a bubble, traveling along the cuvette, crosses the readout light beam, radiation is strongly scattered and a characteristic fingerprint shape of the photo-detected signals versus time is clearly observed. Experimental testing proves that air bubbles can be successfully detected and counted. Their traveling speed can be estimated while simultaneously monitoring the refractive index of the fluid.

## 1. Introduction

Detection and count of air bubbles in fluids, as well as the measurement of their size and velocity, is of primary importance in several fields of applications, ranging from the automotive and fuel industries to microfluidics and medicine [1]. In particular, the correct identification of air bubbles is fundamental in medical equipment that delivers fluids to patients’ bodies, such as drug delivery pumps, blood filters, and hemodialysis modules [2].

### 1.1. State of the Art on Acoustic and Electromagnetic Methods for Air Bubble Recognition in Fluids

Several works based on a wide variety of techniques and readout methods for bubble identification can be found in the literature. For example, acoustic methods are widely diffused.

In [3], authors developed an acoustic cylindrical chamber surrounded by a piezoelectric transducer that, simultaneously, generates low-frequency standing pressure waves and measure changes in the resonance frequency of the camber due to the presence of bubbles in the fluid. Yet interesting, this solution requires the dedicated design of a chamber with a high Q-factor.

In [4], Benech et al. presented an ultrasonic sensor with a couple of polymer transducers (a transmitter and a receiver), applied to the pipe where bubbles and liquid flow. In [5] the authors developed a similar system for bubble identification in drug delivery circuits, based on the generation and detection of acoustic waves through a tube where bubbles can flow. However, both solutions ([4,5]) require putting the transducers in contact with the tube and using a complex electronic circuit for signal processing.

In [6], a short study is reported on the use of magnetic resonance imaging of water for locating and visualizing air bubbles inside complex fluid-processing equipment, such as water filters, blood filters, and kidney dialysis modules. However, magnetic resonance requires extremely expensive instrumentation and complex measurements.

Another work demonstrated the detection of air bubbles inside a 6-cm-diameter pipe based on portable mm-wave Doppler radar [7]. The speed of the bubbles was estimated, too. The main drawbacks of this system are that it is very bulky and it can mainly detect large-size bubbles.

In [8], the authors reported a three-electrode capacitive sensor structure, which was fabricated by PCB technology, to monitor air bubbles in a fluidic channel.

A complex system based on the combination of ultrasonic transmission tomography and electrical resistance tomography for imaging bubbles in two-phase gas/liquid was presented in [9]. Another sensing device based on reflection-mode ultrasonic tomography for the measurement of rising gas bubbles is described in [10]. However, these works ([9,10]) are based on the use of very bulky pipes with a height of about 30 cm and diameter of 5–10 cm and transducers with a dimension of several centimeters: thus they are not suitable for a miniaturized design.

In [11], the authors developed a method based on direct-contact heat transfer. They proposed a hybrid imaging analysis model combining the U-net convolutional neural network with the quantile regression method and empirical mode decomposition for image segmentation and bubble pattern extraction. This method, yet interesting, requires complex steps of image processing and statistical analyses and it is not suitable for real-time bubble identification.

In [12], bubbles inside a dielectric tube are irradiated by a microwave generator and the microwave nearfield is visualized through a thermoelastic optical indicator microscope. This work needs a complex integration of microwave and optical technologies to detect air bubbles as well as the use of expensive components, such as a CMOS camera and a liquid crystal retarder.

### 1.2. State of the Art on Optical Methods for Air Bubble Recognition in Fluids

Optical-based solutions for air bubbles recognition in fluids are particularly interesting since they are not subject to electronic interferences and make use of non-ionizing radiation.

For instance, in [13,14], the authors developed a laser tomography system to detect bubbles inside a vertical column pipeline. Sixteen laser pointer-photodetector couples are mounted in a jig around the circumference of the pipe. The voltage signals generated by the detectors are acquired and fed to a back-projection algorithm, able to reconstruct the tomographic images. From image analyses, it is possible to detect the bubbles and estimate their diameter. However, the system was demonstrated only with large bubbles a with diameter of around 2 cm and the achievable spatial resolution looks quite poor.

Jamaludin et al. proposed a similar optical tomography configuration based on four laser diodes and eight CCD sensors to detect air bubbles in crystal-clear water [15]. Here, the acquired data allow to retrieve the crossing events through image reconstruction based on the filtered image of linear back projection with hybrid algorithms. The main limitations of this work are the complex geometry and the tricky lens expansion system.

In another work [16], the authors realized a dip-in optical probe with an LED and a CCD camera. This patented solution is based on an invasive approach because the probe needs to be immersed in the fluid.

In [17], the authors proposed two setups for optical counting and sizing of air bubbles in a clear blood analogue sample. In the configuration for bubble counting, a laser light is shone onto a transparent cuvette and scattered radiation is captured by means of a camera located at 30° with respect to the incident beam. On the other hand, bubble sizing is achieved by using a shadowgraphy technique, that sizes bubbles based on images of their shadows generated by back illumination with a pulsed laser and acquired with a high-speed camera. The performances of the proposed methods are then compared with those of two commercial instruments based on ultrasounds, revealing better precision and accuracy, in particular for high flow rates. However, the main drawback of this work is that two different optical setups are needed to attain bubble counting and sizing.

In 2020, Alfarraj et al. measured the air bubble size and velocity in diesel using an optical method [18]. They used a 532 nm double-pulse laser (based on Nd:YAG) with cylindrical lenses to expand the 2-D sheet beam impinging on a cuvette and a CCD camera to acquire pictures. They were able to measure number, speed, and size of micro-air bubbles, but Nd:YAG lasers are extremely expensive.

In [19], a fiber optic reflectometer technique based on a single fiber probe is investigated for measuring bubble velocity, diameter, and void fraction in a multiphase flow. The method is based on the interference between the scattered light from the bubble and the Fresnel-reflected light from the tip of the optical fiber.

In [20], Wesley et al. proposed an optical microscopy system constituted by a camera, a LED array and a diffuser for taking high-contrast images of microbubbles in a tank. The use of the bulk CMOS camera does not make the system suitable for integration in healthcare applications.

To sum up, these detection systems and configurations are surely promising but still suffer from some general remarkable drawbacks, such as the need for expensive and sophisticated instrumentation and large dimensions, and miniaturization may not be easily implemented. Moreover, the proposed optical methods often rely on image acquisitions with cameras and complex processing or on invasive probes.

### 1.3. Current Work Motivations

Focusing our attention on healthcare, when a fluid needs to be administered intravenously to a patient, the formation of air bubbles and their insertion in the bloodstream may induce the phenomenon of embolism, which can lead to very dangerous consequences for the patient. Indeed, air bubbles can clog the vessels and prevent blood from flowing in the correct way, causing strokes, heart failures, and pleural effusion, just to cite a few possible negative outcomes. In particular, embolism is actually a complication that can arise when artificial nutrition solutions are administered directly in the bloodstream with an infusion pump to a patient who cannot naturally feed himself [21,22]. Hence, further investigations to design smarter and more compact systems for bubble detection, suitable for integration in fluidic pump, would increase the safety of these medical devices.

In this work, the instrumental configuration that we originally designed only for the recognition of solutions for parenteral artificial nutrition (PAN) [23] is here upgraded to a multi-parameter measuring system able not only to identify transparent fluids through their refractive index (RI) but, at the same time, also able to recognize the presence of air bubbles in the fluidic circuit and measure their flow velocity. As in the previously reported scheme [23], a laser beam travels obliquely through a cuvette and is then back-reflected by a mirror toward a position that depends on the filling fluid RI. The fluid recognition function is accomplished by detecting the displacement of the transmitted spot due to the fluid under test, with respect to a reference fluid, with a 1-D linear position sensitive detector (PSD) [24], an optical detector typically employed, for example, in laser triangulation sensors. The identification of an air bubble is achieved by recognizing a characteristic fingerprint in the PSD photo-detected signals in the time domain. Its peculiar time-dependent line shape is due to a traveling bubble that, intercepting at different instants the incident and reflected laser beams, scatters light and prevents having a well-defined spot on the PSD twice in a short time sequence.

### 1.4. Paper Structure

Here, we show an optical detection system that is suitable to distinguish fluids with different refractive indices and to identify single traveling bubbles, also retrieving their speed. In Section 2, the instrumental configuration is described in detail. Section 3 presents the principle of operation of the system as a refractive index sensor and as an air bubble detector. Finally, in the conclusions, comparisons with other reported solutions and some future developments are reported.

## 2. Materials and Methods

**Figure 1:**
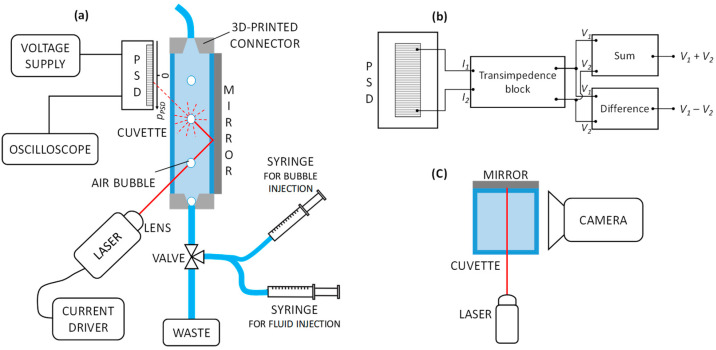
Experimental detection of air bubbles. (**a**) Bubbles injected by means of a syringe interrupt the laser beam twice (front view). (**b**) Block schematic of the PSD signal processing circuit. (**c**) Preliminary setup for bubble detection including a camera (top view).

The instrumental configuration developed in [17] and here exploited for multi-parameter sensing is shown in Figure 1a. 

### 2.1. Fluidic Section

A standard 1 cm × 1 cm square section polystyrene cuvette (Cheimika S.A.S, Capezzano Inferiore, Italy), traditionally used in spectrophotometers, with length of 5 cm, is used as a fluidic reservoir. The cuvette was customized by removing the bottom and by adding, on both top and bottom, 2 3D-printed connectors for fastening plastic tubes. A 3-port fluidic valve is used to connect the bottom connector to a waste container, a syringe for fluid injection, and a syringe for bubble generation. 

### 2.2. Optical Readout

Readout radiation is provided by a semiconductor laser (HL6748MG, Thorlabs, Newton, NJ, USA) with emission wavelength at 670 nm and maximum optical power of 10 mW, powered by a bench current driver (LDC 500, Thorlabs, NJ, USA). The laser current was set above the threshold, around 24 mA. A glass lens (LTN330-B, Thorlabs, NJ, USA) with a focal length of 3.1 mm and numerical aperture of 0.68 is placed in front of the source and the light beam is focused to obtain the smallest light spot onto the surface of the detector. The light beam is shined onto 1 side of the fluidic reservoir with an angle of incidence of 45°. Radiation crosses the cuvette containing the fluid twice, bouncing once on an Al-coated mirror (ME1S-G01 Thorlabs, NJ, USA), attached to the cuvette on the opposite side with respect to the laser. 

### 2.3. Optical Signal Detection and Processing

After crossing the fluid, the light beam reaches a 1-dimensional high linearity PSD with an onboard signal processing circuit (1L10-SU74-SPC02, by SiTek Electro Optics AB, Partille, Sweden) placed parallel to the cuvette, on the same side of the laser, a few millimeters far from it. The PSD active surface is 10 mm (*L_PSD_*) × 2 mm. The detector is powered with a ±15 V supply (PS283, Tektronix, Beaverton, OR, USA). 

The selected photodetection component by SiTek integrates within the same device both the PSD and all the required analog electronic circuits that generate two output voltage signals. Whereas the difference voltage signal output, *V*_1_ − *V*_2_, carries information about the position of the light spot along the sensor area, the sum signal *V*_1_ + *V*_2_ is linearly proportional to the total optical power reaching the detector. In more detail, the photocurrents generated at both ends of the PSD active region are first converted in voltage (*V*_1_ and *V*_2_) by transimpedance amplifiers. Additional analog circuits, directly integrated in the device for onboard signal processing, provide difference voltage signal (*V*_1_ − *V*_2_) and sum voltage signal (*V*_1_ + *V*_2_) as ready-to-use output signals (Figure 1b). The voltage output signals provided by the SiTek component are visualized in real-time and acquired with an oscilloscope (MDO3034, Tektronix, OR, USA) with a sampling frequency of 10 kHz. Eventually, data are processed in MATLAB environment.

As shown in Figure 1c, during preliminary testing for bubble identification, a grey-scale CMOS camera (FLIR Grasshopper GS3-U3-41C6NIR-C, FLIR Systems, Wilsonville, OR, USA) was positioned at 90° with respect to the plane of the incident beam, looking at the free side of the cuvette. The camera is here used only to record videos for witnessing the passage of the air bubbles in the fluidic channel. The frame sequence (acquired with framerate of 30 fps) is synchronized with the PSD output to correlate the recorded events with the characteristic fingerprint of the photo-detected signals in time domain. It is important to underline that the camera is not meant to be included in the final configuration, since actual bubble detection relies only on the analysis of the signals provided by the PSD.

## 3. Results

### 3.1. Refractive Index Detection

Here we briefly recall the principle of operation of the scheme reported in Figure 1 for fluid distinction by means of refractive index measurements applied to analyze water-based solution. Indeed, as mentioned, the selected photodetection component by SiTek, with onboard signal processing, integrates within the same device both the PSD and all the required analog electronic circuits that generate two output voltage signals. Whereas the difference voltage signal *V*_1_ − *V*_2_ carries information about the position of the light spot along the sensor area, which depends on the RI of the fluid in the cuvette, the sum signal *V*_1_ + *V*_2_ is linearly proportional to the total optical power reaching the detector.

**Figure 2:**
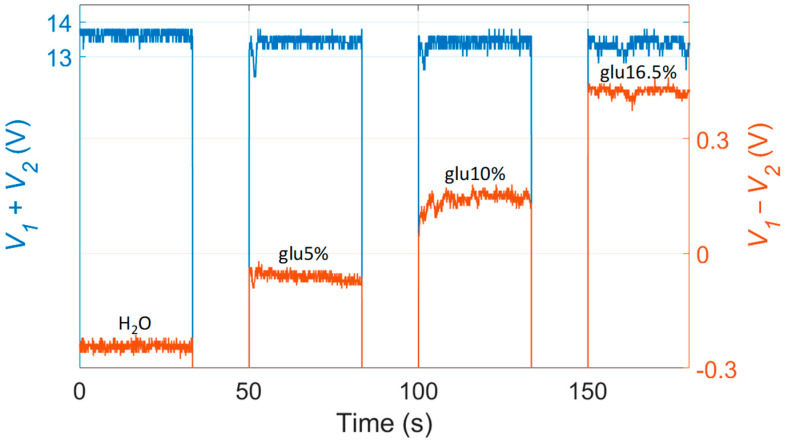
Experimental results obtained during testing of water-glucose dilutions with different concentrations and RI. The sum signal (blue trace) has an almost constant value for every sample; on the other hand, the difference signal (orange trace) keeps changing because of the light beam displacement occurring when the RI of the fluid in the cuvette increases.

During preliminary testing of the multi-parameter measuring system, the cuvette was sequentially filled with different water-glucose dilutions with concentration from 0% (water) up to 33%, thus with RI in the interval 1.3325–1.3788 RIU, and emptied each time between different samples. All measurements were carried out at a constant room temperature of 25 °C with fluctuations of about ±0.5 °C, which do not affect the fluid RI. Experimentally detected sum and difference signals relative to a filling sequence of four different fluids are reported in Figure 2. The sum signal (blue trace) switches between its top value, approximately 13.7 V, when the water-glucose solution is filling the cuvette, and a much lower value when the cuvette is emptied: in this situation, the output spot moves outside the PSD active surface since light traveling direction is changed by the sudden drop of refractive index. In other words, the two-level sum signal indicates the presence or absence of a water-based transparent fluid in the cuvette. The position *p_PSD_* of the spot on the PSD is directly proportional to the difference signal *V*_1_ − *V*_2_ (orange trace of Figure 2) since.

*p_PSD_* = (*L_PSD_*/2) × (*V*_1_ − *V*_2_)/(*V*_1_ + *V*_2_) (1)

where *L_PSD_* = 10 mm. When *V*_1_ − *V*_2_ = 0, the spot is in the center of the photosensitive active region; negative values of *V*_1_ − *V*_2_ indicate that the spot is moving toward the upper end of the PSD, whereas positive values of the same signal indicate that the spot is moving toward the lower end of the PSD, that is closer to the input position of the readout beam (see the deployment of the devices in Figure 1). As shown in Figure 2, the difference signal assumes different values for increasing RI values due to more and more concentrated glucose solutions (from slightly negative to positive values). When the cuvette is emptied, the difference signal suddenly drops to a negative value, since the output spot is projected toward the upper end of the PSD and eventually moves outside the PSD active surface.

By converting the sum and difference signal, only considered when a fluid (not air) is in the cuvette, in spot displacement *p_PSD_*, using Equation (1), the linear calibration curve of the system, as volume RI sensor, was retrieved yielding a sensitivity of 13960 µm/RIU, in agreement with the detailed theoretical simulations presented in [23] describing the path traveled by the laser beam inside the cuvette. More details about a specific application of the RI measuring system for PAN recognition were reported in [23].

In particular, the RI resolution [25] of the sensor (that is the minimum RI variation that can be detected) is around 0.0005 RIU. The maximum RI difference (with respect to a reference fluid) that can be measured is related to the length of the PSD sensing region. Indeed, the maximum detectable RI difference is the one that produces the maximum light spot displacement that can be measured in the linear region of the PSD (8 mm over a total length of 10 mm). Moreover, it must be taken into account that the PSD response is not linear in a very wide range of RI values but the sensitivity tends to decrease as the RI variation increases. In view of all these considerations, the theoretical model described in [23] allowed to estimate a maximum range of measurable RI variation of the order of 1.67 RIU. 

### 3.2. Air Bubble Detection

The possibility to identify air bubbles by means of the measuring scheme shown in Figure 1 was then carefully investigated. Experimental tests were performed with the cuvette filled with deionized water and in static conditions, i.e., with the fluid still inside the channel. Several air bubbles were pushed inside the fluidic circuit using the syringe attached to the valve for this aim. Bubbles crossing the cuvette have a diameter of approximatively 2–3 mm: their size is mainly related to the dimension of the connector that air bubbles are generated from.

**Figure 3:**
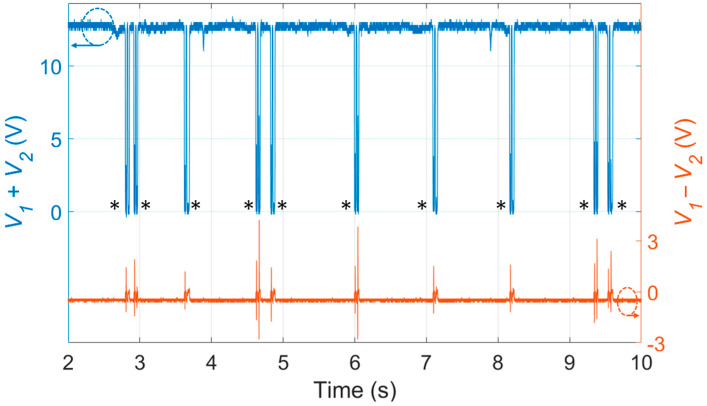
Experimental results obtained during testing of air bubbles: sum signal (blue trace) and difference signal (orange trace) generated by the PSD. Every time an air bubble interrupts the light beam, both signals exhibit a very peculiar shape: each black asterisk indicates the passage on one bubble. The dashed circles with the arrow indicate the reference variable.

Several experiments were carried out and the signals provided by the PSD, when an air bubble crosses the laser beam, were analyzed. Figure 3 reports the photo-detected signals acquired when a sequence of 10 bubbles was generated in a controlled way (blue trace: sum signal; orange trace: difference signal). By watching the video, recorded simultaneously by the camera, 10 bubbles were clearly observed: all of them are correctly identified by the PSD signals.

### 3.3. Characteristic Fingerprint Shape of the Photo-Detected Signals 

We must discuss the characteristic fingerprint shape of the photo-detected signals. When the cuvette is filled with water, without bubbles, the sum signal reaches its maximum value, whereas the difference signal is slightly negative, in accordance with the results shown in Figure 2. When an air bubble crosses the laser beam, light is scattered and diffused all around: hence, the optical power density reaching the PSD temporarily becomes extremely small and both sum and difference signals jump to 0 V, exhibiting a fast transition. Indeed, the signals shown in Figure 3 exhibit a total of 10 switching (indicated by the black asterisks), each of them corresponding to a bubble transit.

**Figure 4:**
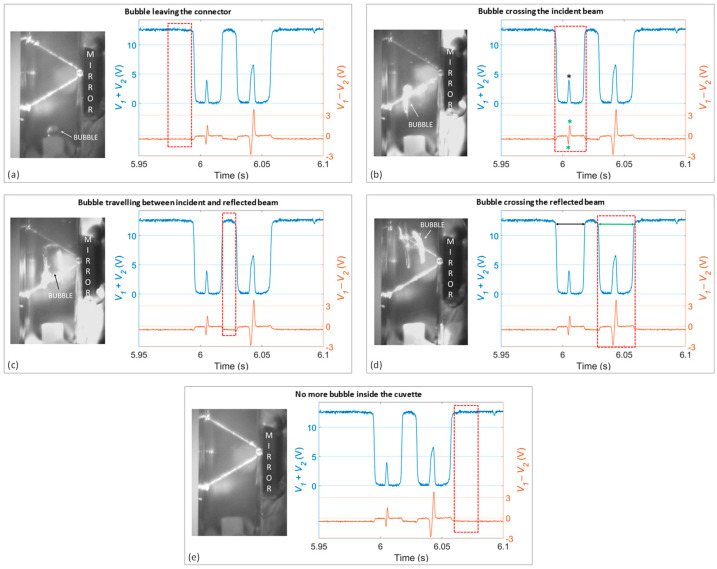
Experimental results relative to air bubble detection generated by the PSD: sum signal (blue trace) and difference signal (orange trace). Red rectangles in each subset indicate the signal portions corresponding to the frames on the side. Every time an air bubble interrupts the light beam, both signals exhibit very peculiar spikes. In particular, the pictures and the PSD signals of five subsets illustrate, respectively, the following steps: (**a**) the air bubble is generated, and it is about to leave the connector, (**b**) the bubble crosses the incident beam (the black asterisk indicates the local peak of the sum signal due to the crossing of the central part of the bubble; the green asterisks indicate the double-negative-positive peak transition of the difference signal due to the crossing of the central part of the bubble), (**c**) the bubble is travelling between the incident and reflected beam, (**d**) the bubble crosses the reflected beam: black and green arrows represent the time required to bubble for crossing the incident and reflected beam, respectively, (**e**) and the bubble has left the cuvette.

Moreover, it is particularly interesting to zoom the *V*_1_ − *V*_2_ and *V*_1_ + *V*_2_ signals generated during a single bubble crossing and carry out a one-to-one comparison with the corresponding video frames recorded by the camera. Figure 4 shows the step-by-step analysis of the bubble passage in the cuvette: each frame allows to visualize the different positions of the bubble with respect to the laser beams and corresponds to the portion of signal highlighted by the dotted red rectangles. In particular, the graphs reported in Figure 4a–e are zoomed views of a short time interval of the graphs presented in Figure 3 and they correspond to the passage of a single air bubble. It can be noted that what in Figure 3 looked like a single spike in the sum and difference signals is composed of two double transitions since the bubble crosses the light beam traveling to the mirror and then the beam is reflected by the mirror.

By following the sequence in more detail, Figure 4a shows the situation in which an air bubble generated by the syringe is leaving the connector and entering the cuvette. Both beams (to and from the mirror) are not interrupted by the bubble yet and thus *V*_1_ + *V*_2_ and *V*_1_ − *V*_2_ take the expected values due to the presence of water in the cuvette.

Afterwards, the bubble starts crossing the channel and it first encounters the beam traveling to the mirror (Figure 4b). As highlighted by the red rectangle, the sum signal drops to 0 V, it stays constant at this value for a few ms. It then exhibits a narrow, low peak (indicated by the black asterisk) reaching an intermediate positive value. It drops to 0 V again for a few ms and, finally, jumps back to the top value. The difference signal has a different shape. It also jumps to 0 V synchronously with the sum signal, but it then features a double-negative-positive peak transition, going from −1.3 V to 1.5 V (indicated by the green stars). The described behavior of the sum and difference signals is likely due to the fact that the beam is initially scattered by the bubble (and the sum signal drops to zero), but for a very short time interval, the beam is actually back-reflected by the water-air interface toward the active area of the PSD at a varying angle, which could well explain the negative-positive transient in the difference signal.

Then, as shown in Figure 4c, the bubble travels between the incident and reflected laser beams, which therefore are not crossed. The sum and difference signals assume the standard values corresponding to the static situation in which the light beam is simply travelling in water and does not go through the bubble, as already stressed in Figure 4a.

Then, the air bubble crosses the laser beam reflected by the mirror (Figure 4d). In this situation, the shape of the signals provided by the PSD is fully comparable to what was observed when the bubble was crossing the beam toward the mirror (Figure 4b). The time required for the second beam crossing (indicated by the green arrow in Figure 4d) is longer than that required for the first crossing, indicated by the black arrow in Figure 4d (29.2 ms vs. 24.1 ms). This delay could be explained by the observation that the air bubble seems to undergo a slight elongation (that can also be appreciated in the reported video frame in Figure 4d), thus more time is necessary to fully cross the laser beam.

Eventually, the bubble keeps moving towards the upper part of the cuvette thus exiting from the region illuminated by laser light (Figure 4e). Hence, the sum and difference signals jump back to the initial values, typical for a homogeneous fluid such as water. The peculiar shape of the sum and difference signals was reproducible and observed for all the bubbles detected by the camera and the PSD, representing a characteristic fingerprint of their crossing.

### 3.4. Estimation of Air Bubble Traveling Speed

**Figure 5:**
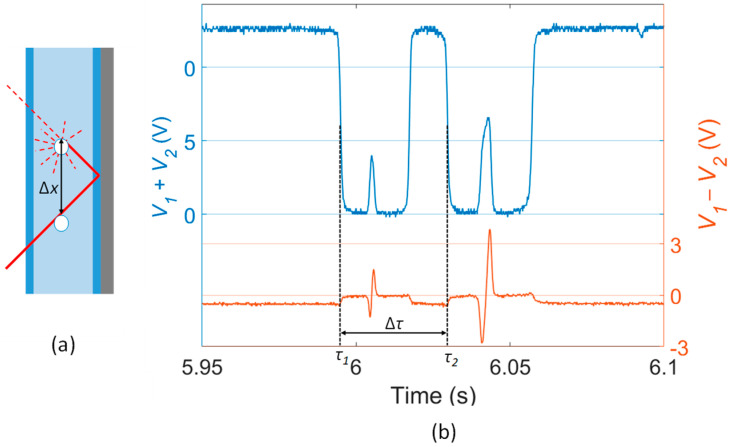
Calculation of air bubble velocity. (**a**) Distance Δ*x* travelled by the bubble between the incident and reflected beams. (**b**) Calculation of the time interval Δ*τ*.

With the proposed system, it was also possible to retrieve the velocity of the air bubble (Figure 5). First, the distance Δ*x* between the incident and reflected laser beams (indicate by the black arrow in Figure 5a) was retrieved, supposing the bubble crossing the cuvette at its center. By knowing the angle of propagation of light in the channel and the width of the cuvette, we attained Δ*x* = 0.502 cm. Then, the time interval Δ*τ* = *τ_1_* − *τ_2_* necessary to travel the distance Δ*x* was retrieved from the analysis of the *V*_1_ + *V*_2_ signal. In particular, the values of *τ_1_* and *τ_2_* were retrieved at the time when *V*_1_ + *V*_2_ drop to half of its top value (Figure 5b), resulting in a Full Width at Half Maximum of the switching Δ*τ* = 0.0348 s. Then, the bubble speed was retrieved as *v* = Δ*x*/Δ*τ* = 14.4 cm/s.

Then, for further validation of the result, the computation was repeated considering the video acquired by the camera and the Δ*τ* between the 2 frames showing the bubble crossing the incident and reflected beams, which yielded Δ*τ* = 0.0342 s and *v* = 14.7 cm/s, thus in accordance with the results obtained processing the photo-detected signal. In principle, also faster bubbles could be detected. Indeed, the PSD response to the bubble crossings is almost instantaneous and the maximum speed of the detectable bubbles mainly depends on the slew rate of the operational amplifier embedded on the PSD board and on the sampling frequency of the oscilloscope used for signal acquisition. 

## 4. Conclusions

Correct identification of air bubbles is fundamental in many medical applications where fluids are administered intravenously. Indeed, bubbles flowing in blood vessels would induce hazardous consequences for a patient’s health.

In this work, we have demonstrated a multi-parameter measuring system that can simultaneously detect air bubbles traveling in transparent fluids and distinguish fluids based on their refractive index by exploiting the same instrumental configuration, preliminary applied for the recognition of commercial solutions for parenteral artificial nutrition [23]. While recognition of solutions is carried out by measuring the light beam displacement on the PSD surface due to their different RI, simultaneous identification of air bubbles inside the cuvette is possible thanks to the fact that each of them interrupts twice the laser beam, providing a fingerprint signal. Experimental measurements performed in water demonstrated that single air bubbles can be correctly identified and counted. Moreover, thanks to the presence of the double light beam, it was possible to successfully retrieve the bubble velocity by considering the time interval between the intersection of the incident and of the reflected laser beam. The results presented in this paper are extremely promising and they suggest the possibility of integrating this multi-parameter measuring system in medical devices managing fluids that enter patients’ bodies such as dialysis modules, blood filters, and infusion pumps. Indeed, our sensing platform could be inserted along the external fluidic path of these medical instruments to monitor the RI of fluids and detected the presence of dangerous air bubbles generating along the fluid stream connected to the patient’s body.

Compared to other works reported in the literature based on ultrasounds or capacitance measurements, our optofluidic configuration does not require the design of dedicated pipes or special geometry and it has not to be in direct contact with the tubes containing the liquid, thus being suitable for remote monitoring. Moreover, compared to other optical detection systems, our configuration is very compact and suitable for miniaturization and could be implemented even using low-cost optical and fluidic components, making the sensing platform suitable to be used also for homecare applications. It is worth stressing that the simple detection method of RI and bubbles makes this multi-parameter platform particularly suitable for real-world industrial applications.

Future work could be devoted to further signal processing to extract information about the size of the bubbles from the voltage signal shape. Also, a digital algorithm could be implemented for the automatic counting of bubbles. Eventually, this sensing platform could be exploited for the analysis of different types of particles inside fluids.

## Data Availability

The data presented in this study are available on request from the corresponding author.
